# New Records of Symbiotic Amphipods on Red King Crabs in the Coastal Barents Sea

**DOI:** 10.3390/biology15020160

**Published:** 2026-01-16

**Authors:** Alexander G. Dvoretsky, Vladimir G. Dvoretsky

**Affiliations:** Murmansk Marine Biological Institute of the Russian Academy of Sciences (MMBI RAS), Murmansk 183038, Russia

**Keywords:** epibiosis, *Paralithodes camtschaticus*, amphipod, Barents Sea

## Abstract

Monitoring epibiotic communities on the invasive red king crab (*Paralithodes camtschaticus*) in the Barents Sea is crucial for understanding the co-adaptation between this species and the local benthic fauna. Red king crabs were collected during regular diving surveys conducted in the coastal Barents Sea in 2015, 2021, and 2022. A detailed examination revealed the presence of two amphipod species, *Metopa pusilla* and *Crassicorophium bonellii*, which were not previously documented as epibionts on this host. With these additions, the total number of epibiotic amphipods on Barents Sea red king crabs rises to nine species (versus two in the native Sea of Okhotsk). Amphipod colonization was skewed toward large males, likely reflecting their greater migratory behavior. The prevalence of *Metopa pusilla* ranged from 1.9% to 4.3%, with a mean intensity of one individual per infested crab; *Crassicorophium bonellii* exhibited prevalence of 4.7–14.3% and mean intensity of 1.3–3.3 individuals. The primary colonization sites were the carapace and limbs. Given the low infestation parameters and the epibionts’ localization away from critical structures like the gills and egg clutches, it is concluded that these amphipods pose a negligible risk to host health.

## Simple Summary

The red king crab is a large, commercially significant decapod that has been introduced into the Barents Sea to enhance regional fishery yields. In its invasive range, this host has become colonized by a diverse epibiotic community. Here, we report two novel amphipod epibionts (*Metopa pusilla* and *Crassicorophium bonellii*) on red king crabs in the Barents Sea. These records expand the known biodiversity of crab-associated amphipods in this region and provide some biological data on each symbiont. Both species exhibited low prevalence and intensity of infestation, and their attachment sites (limbs and carapace) suggest minimal risk to host health.

## 1. Introduction

Epibiotic associations—interactions between a basibiont (host) and an epibiont attached to the host’s external surface—are ubiquitous in marine ecosystems, as these facultative partnerships often confer advantages to at least one participating organism [1,2]. These associations are of significant scientific interest as they involve a wide variety of ecologically and economically important species [3]. In the Barents Sea, a prominent example is the red king crab, *Paralithodes camtschaticus* (Tilesius, 1815). This large crustacean, prized for its meat quality [4], was introduced in the 1960s to establish a new commercial fishery [5]. This initiative aimed to utilize the region’s high productive potential both in terms of primary production [6] and fishery productivity [7], driven by inflows of warm Atlantic water [8], in an area otherwise lacking commercially significant crab species [9]. The introduction has been successful [10], and the red king crab now constitutes a major fishery in its non-native range [11]. For instance, in 2022, 2023, and 2024, the annual catch of red king crab in Russian waters of the Barents Sea accounted for landings accounting 12,529, 10,420, and 12,468 metric tons [12,13].

Like many invasive species, the red king crab has substantially expanded its distribution in the Barents Sea through a combination of adult migration and larval dispersal [14]. It has achieved high abundances in both coastal and open-sea areas [15,16,17,18,19], raising concerns about its impact on native benthic communities [20,21,22]. Recent research indicates that the crab can alter the structure and composition of coastal habitats through its feeding and burrowing activities [23,24,25,26,27,28]. A further important consideration is the colonization of the crab’s exoskeleton and gill chambers by various benthic organisms, both sessile and mobile [29,30,31,32,33,34]. Long-term monitoring has demonstrated that migratory crabs facilitate the regional dispersal of associated symbionts such as amphipods and fish leeches, thereby reshaping local benthic assemblages [33,35]. Additionally, such studies yield novel data on the biology of symbiotic organisms [36,37,38,39,40,41,42], which can be particularly valuable for species that are difficult to sample using conventional methods [43,44,45,46].

The aim of the present study is to document the occurrence of two amphipod species, not previously recorded as epibionts, on red king crabs in the coastal Barents Sea. This finding contributes to understanding the formation of novel symbiotic relationships in the context of biological invasions.

## 2. Materials and Methods

**Figure 1:**
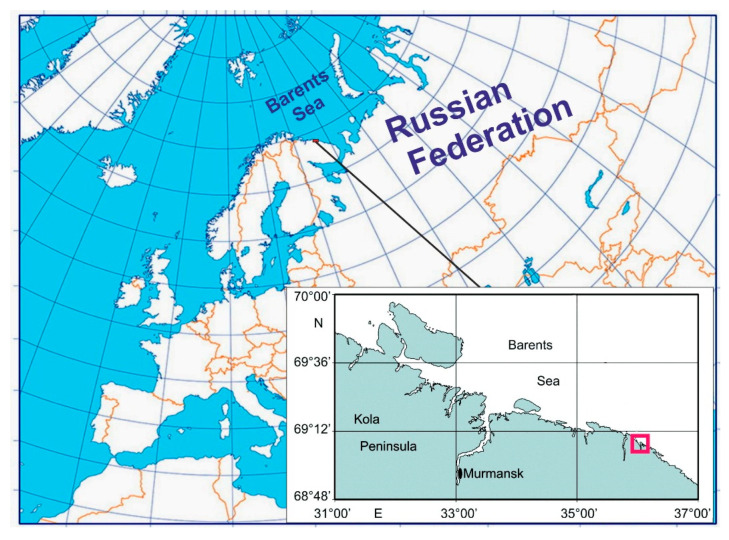
Map of the study area showing the location of Dalnezelenetskaya Bay (red square) in the coastal Barents Sea.

Specimens of the red king crab were collected in Dalnezelenetskaya Bay (Figure 1), a semi-enclosed gulf separated from the open Barents Sea by five islands.

Tidal amplitudes in this bay reach up to 4.2 m, i.e., considerably lower than in adjacent open habitats [47]. While maximum depths reach 200 m, the mean depth ranges from 7 to 18 m. The site is characterized by a variable hydrological regime [48]: surface water temperature fluctuates seasonally from 0.7 °C in February to 9.7 °C in August, and salinity ranges from 32.2 to 34 psu [49]. The predominant substrates are small stones, gravel, and silty sand [50].

SCUBA diving surveys were conducted in July 2015, 2021 and 2022 at depths of 5–40 m. In each survey, a standardized transect grid consisting of 12 transects was employed in sample areas exhibiting the highest crab abundance. All collected crabs were transported live to the coastal laboratory for immediate examination. For each host crab, the following data were recorded: sex (determined visually by the morphology of the abdominal flaps), carapace width (CW; measured as the greatest straight-line distance between the lateral spines using calipers), and shell condition (categorized visually according to the scale proposed by Donaldson and Byersdorfer [51]).

Each crab was examined for epibionts following a standard protocol established in our previous studies [33,35]. The host’s body was divided into five sections for systematic inspection: carapace, limbs, abdomen, mouthparts, and gills. The gill chambers were examined after the dissection of the crab. All associated epibionts were carefully removed, fixed in a 4% formaldehyde solution, and subsequently transported to the laboratory in Murmansk for identification. Amphipods were identified to species level using an MBS-10 stereomicroscope (Lytkarinsky Optical Glass Plant, Lytkarino, Moscow region, Russia) and standard taxonomic keys [52]. The current taxonomic nomenclature was verified against the World Register of Marine Species [53].

Epibiotic infestation was quantified by calculating (1) prevalence, defined as the percentage of crabs colonized by each associate species, and (2) intensity, defined as the mean number of epibiont specimens per colonized host [54].

Percentages (sex ratio, prevalence, occurrence of colonized crabs at different depths) were analyzed using Chi-square tests, while quantitative data (crab size) were compared using non-parametric Kruskal–Wallis tests, as the data failed assumptions of normality [55]. All mean values are presented alongside their standard errors (mean ± SE).

## 3. Results and Discussion

A total of 250 red king crabs were collected during the study period (2015: n = 53; 2021: n = 127; 2022: n = 70). The sampled population was predominantly composed of mature individuals, with proportions of 92.5% (2015), 97.3% (2021), and 98.6% (2022), yielding a combined maturity prevalence of 94.8%. The sex ratio was significantly skewed towards females in 2015 (2.8:1; χ^2^ = 11.79, df = 1, *p* < 0.001) and 2021 (5.4:1; χ^2^ = 59.59, df = 1, *p* < 0.001). In contrast, a balanced 1:1 sex ratio was observed in 2022 (χ^2^ = 0, df = 1, *p* = 1). This demographic composition is consistent with previous findings in this coastal area, where a shift towards larger individuals has been documented since 2010, attributed to higher juvenile mortality resulting from significant temperature fluctuations [49]. The female bias in summer samples reflects their lower migratory activity compared to males [56]. The balanced sex ratio in 2022 is likely associated with favorable climatic conditions—such as elevated water temperature and nutrient concentration—that promoted population growth, improved feeding opportunities for males in coastal waters, and their active migration from deeper habitats [13,57].

**Figure 2:**
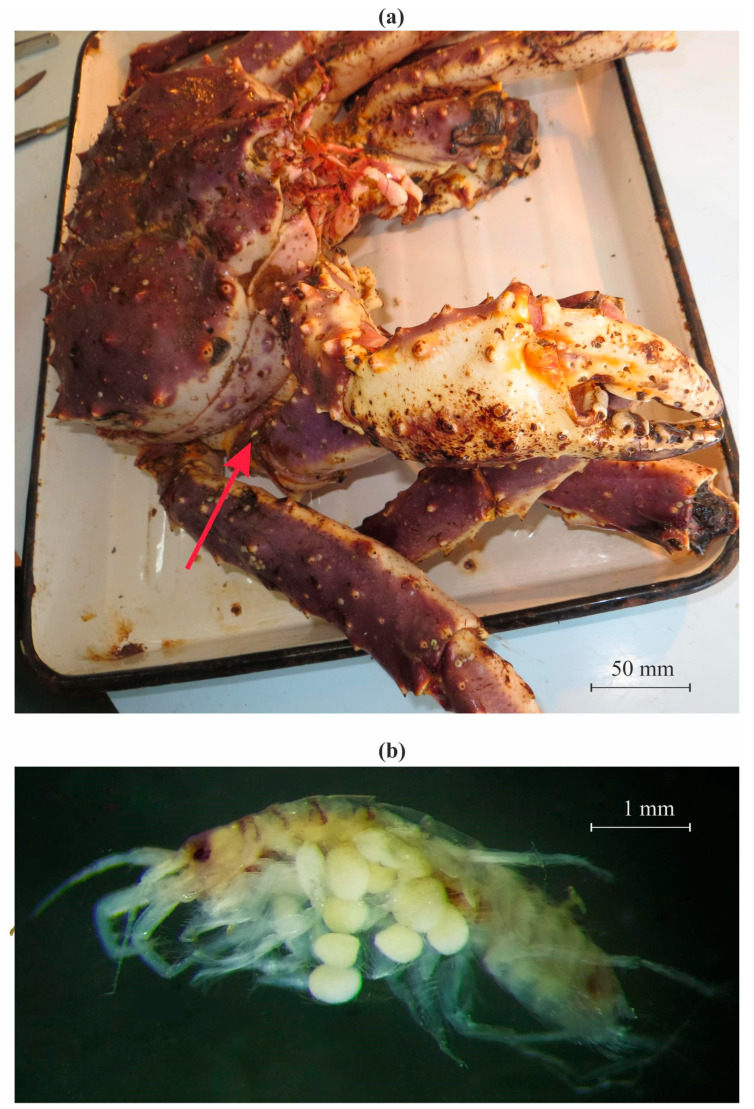
Photographs of a male red king crab, *Paralithodes camtschaticus*, hosting the amphipod *Crassicorophium bonellii* from Dalnezelenetskaya Bay. (**a**) The crab and amphipod (indicated by arrow) located on the host’s pereiopod. (**b**) Close-up view of the egg-bearing female *Crassicorophium bonellii*. Scale bars are provided.

During our surveys, two amphipod species were recorded on *Paralithodes camtschaticus* for the first time: *Metopa pusilla* Sars, 1892 and *Crassicorophium bonellii* (Milne Edwards, 1830) (Table 1; Figure 2).

*Metopa pusilla* has a wide distribution in European shallow waters (down to 50 m), ranging from the North Sea (English and Danish waters) and Skagerrak to northern Norway. It is also present in the Barents Sea, off the east coast of Greenland, and around Franz Josef Land [52]. *Crassicorophium bonellii* is a highly widespread species found along European coasts from Novaya Zemlya to the Mediterranean and Black Seas, along the Atlantic coast of the Americas, and noted off the coast of Chile. It typically inhabits shallow-water kelp forests and clean sands at depths of 2–20 m [52].

Prior to this study, the following amphipod species had been reported as epibionts on red king crabs in the Barents Sea [33,35,59]: *Ampelisca* sp., *Caprella septentrionalis* Krøyer, 1838, *Gammarellus homari* (J.C. Fabricius, 1779), *Ischyrocerus anguipes* Krøyer, 1838, *Ischyrocerus commensalis* Chevreux, 1900, *Ischyrocerus latipes* Krøyer, 1842, and *Ischyrocerus megacheir* (Boeck, 1871). With the addition of the two species reported here, the list of amphipods known to form associations with red king crabs in the Barents Sea now stands at nine species. For comparison, only two amphipod species, *Ischyrocerus commensalis* (prevalence: 49%, mean intensity: 10 specimens per crab) and *Caprella ungulina* Mayer, 1903 (prevalence: 3%, mean intensity: 6 specimens per crab), have been recorded as epibionts of this crab in its native area, Sea of Okhotsk [60].

Detailed data for the crabs with the newly recorded amphipods are summarized in Table 2.

**Figure 3:**
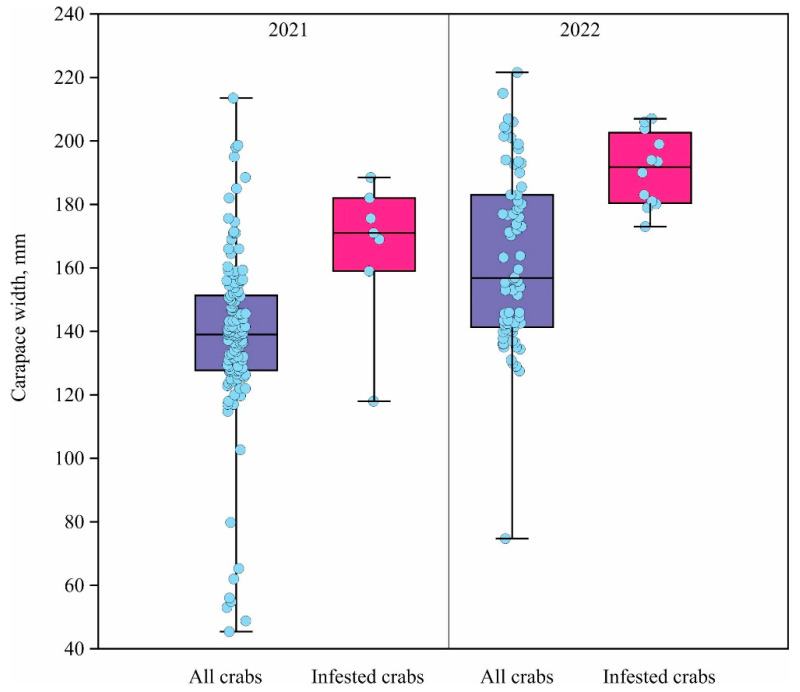
Carapace width (mm) of red king crabs sampled in Dalnezelenetskaya Bay. Box-plots compare the size distribution of all captured crabs versus those colonized by symbiotic amphipods (*Metopa pusilla* and *Crassicorophium bonellii*) in 2021 and 2022.

Depth did not influence the occurrence of symbiotic amphipods, as colonized crabs showed no tendency to be associated with a specific depth range. A comparison between depths of less than 15 m, 15–20 m, and greater than 20 m revealed no significant differences (χ^2^ = 5.44, df = 2, *p* = 0.066). Statistical analysis revealed a significant tendency for both amphipod species to colonize larger crabs (Kruskal–Wallis test: 2021, H = 7.24, df = 1, *p* = 0.007; 2022, H = 11.58, df = 1, *p* = 0.001; Figure 3).

This pattern is likely attributable to the greater surface area available for colonization on larger hosts, a phenomenon commonly observed in marine symbiotic relationships worldwide [45,61,62,63,64,65,66,67,68].

Amphipods were found predominantly on male crabs (18 males vs. 2 females; χ^2^ = 12.81, df = 1, *p* < 0.001). In contrast, the shell condition of the host had no significant effect on epibiont occurrence (χ^2^ = 1.57, df = 2, *p* = 0.486). This male bias aligns with previous studies and can be explained by the larger average size and greater body dimensions of male crabs [69,70], as well as their higher migratory activity, which may increase encounter rates with potential symbionts [56].

The five specimens of *Metopa pusilla* collected consisted of four females (body length 2.1–2.6 mm) and one male (2.0 mm). These sizes are slightly below the typical body length of 3 mm reported for this species in the literature [52,71]. A total of 41 specimens of *Crassicorophium bonellii* were recorded, comprising 7 juveniles (0.9–3.3 mm, mean ± SE: 2.3 ± 0.3 mm) and 34 females (2.7–6.0 mm, 3.6 ± 0.1 mm). The sizes of the females align with previous reports, where lengths of up to 4 mm [58], 6 mm [52], and 5.5 mm [71] have been documented. The pronounced female bias observed is a known characteristic of *Crassicorophium bonellii* populations [52,58].

An association with a large, mobile host like the red king crab offers several potential advantages for amphipods. The defensive behavior of adult crabs against conspecifics and predators [72] provides the epibionts with indirect protection. Furthermore, the extensive migratory movements of the crabs [73] facilitate the dispersal of amphipods across large areas. The host may also serve as a source of food in the form of food remnants, detritus, or other epibionts, a benefit previously documented for other amphipod symbionts (e.g., *Ischyrocerus commensalis* and *Ischyrocerus anguipes*) on red king crabs in the Barents Sea [30,32,40].

Detrimental effects on red king crabs from amphipod epibionts are typically associated with high infestation intensities, particularly when symbionts colonize sensitive areas such as egg clutches [74,75] or gill chambers [32]. However, both *Metopa pusilla* and *Crassicorophium bonellii* exhibited low prevalence and mean intensity (Table 3). It should be noted that the prevalence of *Metopa pusilla* did not differ significantly over the study period (χ^2^ = 2.82, df = 2, *p* = 0.244). In contrast, for *Crassicorophium bonellii*, we observed a significantly lower prevalence in 2021 compared to 2022 (χ^2^ = 4.24, df = 1, *p* = 0.039), most likely due to a higher occurrence of old- and very-old-shelled crabs in 2022 (Table 2, χ^2^ = 11.43, df = 1, *p* = 0.003).

Moreover, these amphipod species were found predominantly on the carapace and limbs (75% and 94% of individuals, for *Metopa pusilla* and *Crassicorophium bonellii*, respectively). This suggests that their current level of infestation is unlikely to negatively impact host health.

## 4. Conclusions

This study provides the first documented evidence of the amphipods *Metopa pusilla* and *Crassicorophium bonellii* as epibionts of the red king crab in the Barents Sea, increasing the known number of amphipod symbionts associated with this invasive host in the region to nine. Both amphipod species demonstrated a significant tendency to infest larger, male crabs, correlating with the higher migratory activity of males in the coastal zone. The low infestation indices, combined with their localization on the carapace and limbs, indicate a commensal relationship that is unlikely to adversely affect the host. For the amphipods, this association likely offers benefits including protection from predators, enhanced dispersal capability, and access to food resources. These findings contribute to understanding the ongoing integration of the red king crab into the Barents Sea ecosystem and the formation of novel symbiotic relationships.

## Figures and Tables

**Table 1:** Key morphological characteristics for the identification of the symbiotic amphipods *Metopa pusilla* and *Crassicorophium bonellii* found on red king crabs in the Barents Sea, summarized from Crawford [58] and Gurjanova [52].

Feature	*Metopa pusilla*	*Crassicorophium bonellii*
Color	Greenish white with brown patches	Whitish with lilac and brownish transverse stripes
Antennae	More or less equal, about one-half body length; no accessory flagellum.	Antenna 1: peduncle article 1 shorter than combined length of articles 2 and 3, armed ventrally with three large, straight spines distally, and one or two sharply curved spines proximally; inner margin of article 1 also armed with one to three spines, the proximal one sharply curved and short; flagellum with a maximum of eight articles. Antenna 2: peduncle article 5 usually with two spines; proximal spine may be as large as distal, smaller, or absent.
Gnathopods	Gnathopod I: simple, slender, 5th and 6th segments are linear; the dactyl with short hairs on the inner edge. Gnathopod II: subchelate, moderately powerful in male, smaller in female; propodus broad distally with oblique, almost straight, slightly crenulate palm delimited by small tooth.	Gnathopod I: palm very oblique, with a row of stout spines of which those near the hinder edge of propod are far the largest; dactyl with one small accessory tooth. Gnathopod II: dactyl with two, or more rarely one, accessory tooth.
Uropods	The setae of uropods III are much longer than the basal segment, which bears 2 spines on the inner edge.	Urosome segments fused; sides of urosome hollowed out at the insertion of uropods 1 and 2.
Others	Telson: oval with two or three pairs of dorsolateral spines.	Rostrum: Short, triangular.

**Table 2:** Morphometric and demographic data of red king crabs (*Paralithodes camtschaticus*) colonized by symbiotic amphipods in Dalnezelenetskaya Bay.

Date	Depth, m	Species	Sex	Carapace Width, mm	Shell
9 July 2015	30	*Metopa pusilla*	F	186	Very old
7 July 2021	16	*Crassicorophium bonellii*	M	182	New
7 July 2021	16	*Crassicorophium bonellii*	M	159	New
7 July 2021	16	*Crassicorophium bonellii*	M	169	New
7 July 2021	16	*Crassicorophium bonellii*	M	175.6	New
7 July 2021	16	*Crassicorophium bonellii*	M	171	New
7 July 2021	7	*Crassicorophium bonellii*	F	118	New
6 July 2022	18	*Crassicorophium bonellii*	M	190	Very old
6 July 2022	18	*Crassicorophium bonellii*	M	193.5	Very old
6 July 2022	18	*Crassicorophium bonellii*	M	206	Very old
6 July 2022	18	*Crassicorophium bonellii*	M	183	Very old
6 July 2022	18	*Crassicorophium bonellii*	M	207	Very old
6 July 2022	18	*Crassicorophium bonellii*	M	173	New
12 July 2022	31	*Crassicorophium bonellii*	M	203.8	Old
13 July 2022	15	*Crassicorophium bonellii*	M	180.2	Old
13 July 2022	15	*Crassicorophium bonellii*	M	194	Old
10 July 2021	43	*Metopa pusilla*	M	188.5	Very old
9 July 2022	36	*Metopa pusilla*	M	199	Very old
11 July 2022	31	*Metopa pusilla*	M	181	New
11 July 2022	31	*Metopa pusilla*	M	179	Old

**Table 3:** Infestation indices of the amphipods *Metopa pusilla* and *Crassicorophium bonellii* on the red king crab population in Dalnezelenetskaya Bay across sampling years.

Species	Year	Prevalence	Intensity, Ind. Per Crab			
			Min	Max	X	SE
*Metopa pusilla*	2015	1.9	1	1	1.0	0.0
*Metopa pusilla*	2021	1.6	1	1	1.0	0.0
*Metopa pusilla*	2022	4.3	1	1	1.0	0.0
*Crassicorophium bonellii*	2021	4.7	1	2	1.3	0.2
*Crassicorophium bonellii*	2022	14.3	1	8	3.3	0.9

Note: Min—minimum, Max—maximum, X—mean, SE—standard error.

## Data Availability

The data presented in this study are available on request from the corresponding author (the data are not publicly available due to privacy restrictions).
